# Goat Paratuberculosis: Experimental Model for the Evaluation of Mycobacterium Persistence in Raw Milk Cheese

**DOI:** 10.3390/microorganisms9102032

**Published:** 2021-09-25

**Authors:** Giulia Pagliasso, Alessia Di Blasio, Nicoletta Vitale, Angelo Romano, Lucia Decastelli, Antonio Quasso, Matteo Ricchi, Alessandro Dondo, Paolo Pastorino, Maria Silvia Gennero, Stefania Bergagna

**Affiliations:** 1 The Veterinary Medical Research Institute for Piemonte, Liguria and Valle d’Aosta, Via Bologna 148, 10154 Torino, Italy; giulia.pagliasso@izsto.it (G.P.); adiblasio@aslto3.piemonte.it (A.D.B.); nicoletta.vitale@izsto.it (N.V.); angelo.romano@izsto.it (A.R.); lucia.decastelli@izsto.it (L.D.); alessandro.dondo@izsto.it (A.D.); mariasilvia.gennero@izsto.it (M.S.G.); stefania.bergagna@izsto.it (S.B.); 2 Azienda Sanitaria Locale TO3, S.C. Sanità Animale, 10064 Torino, Italy; 3 Azienda Sanitaria Locale AT, S.C. Sanità Animale, 14100 Asti, Italy; AQuasso@asl.at.it; 4 National Reference Centre for Paratuberculosis, Istituto Zooprofilattico Sperimentale dell’Emilia Romagna e della Lombardia, Sezione di Piacenza-Gariga, Strada della Faggiola 1, 29027 Gariga di Podenzano, Italy; matteo.ricchi@izsler.it

**Keywords:** *Mycobacterium avium* subsp. *paratuberculosis*, challenge test, food safety

## Abstract

*Mycobacterium avium* subsp. *paratuberculosi*s (MAP) is the causative agent of chronic proliferative enteritis found in ruminants, known as paratuberculosis (PTB). The spread of PTB is increasing in countries with advanced animal husbandry practices, leading to significant economic losses. Moreover, a supposed zoonotic role of MAP in Crohn’s disease (CD) in humans has been discussed by the scientific community; however, although the association between MAP and CD has generally been accepted, it is still up for debate if MAP is the main cause of CD, a contributing factor, or merely a commensal organism for the development of CD. The aim of this study was to assess the survival of MAP during the entire production process of a traditional Italian goat’s raw milk fresh cheese, the “Robiola di Roccaverano”, assessing the survival rate and persistence of MAP in the final product. A mix of MAP field isolates from goats of the Roccaverano area and a reference ATCC strain were used to carry out milk in experimental inoculation. Samples of milk, curd and cheese were taken in two consecutive batches of production. Microbiological challenge tests, evaluated by f57-qPCR, showed a significant decrease in MAP charge during the cheesemaking process for both batches, suggesting the productive process has an impact on MAP survival.

## 1. Introduction

Paratuberculosis (PTB), also known as Johne’s disease, is a contagious bacterial disease typical of domestic and wild ruminants, characterized by chronic progressive granulomatous enteritis [1], with consequent severe deterioration of the general conditions of animals and economic losses [2]. Although the disease was first reported in cattle, the infection has also been identified worldwide among small ruminants [3]. Unlike cattle, where clinical signs include profuse watery diarrhea and severe organic deterioration, infections in small ruminants are more insidious, characterized by progressive weight loss, exercise intolerance, and softening of faeces [3,4], which are also common for parasitic infestation [5].

In addition to zootechnical-health concerns, PTB is also a cause for concern in terms of public health. For several years, the hypothesis that *Mycobacterium avium* subsp. *paratuberculosis* (MAP) is a causative agent in the determinism of Crohn’s disease (CD) has been proposed for several years [6,7,8]. Furthermore, recent studies suggest an involvement of MAP in the development of autoimmune diseases such as type-1 diabetes, sarcoidosis, multiple sclerosis and Hashimoto’s thyroiditis in genetically susceptible individuals [8]. However, even if it appears evident that MAP (both DNA or whole cell) is more likely to be found in patients with CD, this does not authorize the establishment of a causal link between MAP and CD. In fact, MAP is widespread in the environment and the increased probability of isolation in patients with CD could also be due to opportunistic colonization of the patient’s intestines [9].

The zoonotic role of MAP has not been confirmed; however, it would be desirable to pursue a precautionary approach in order to evaluate the exposure risk for humans. Humans are exposed to MAP during direct contact with infected animals or indirectly through food contaminated by faecal material [7]. MAP can be carried by water, meat and dairy products; therefore, the food chain is the main source of infection for humans [10,11]. The latter point is of major concern, particularly for young children, since they have an immature immune system and may be at risk of infection by the ingestion of contaminated milk [7]. MAP has been isolated from milk of cows and other ruminants, such as sheep and goats, in both subclinical and clinical phases of PTB. Milk contamination can have both endogenous origins, through the excretion of infected blood-derived macrophages in milk, and exogenous origins, due to faecal contamination during the milking process [9,12]. Mammary contamination occurs because, in the terminal stages, PTB is a systemic disease, and consequently, the likelihood of producing endogenous contaminated milk is greater if the animal is at an advanced stage of infection [13]. Nevertheless, in small ruminants, the mammary excretion seems to be intermittent and not linked to the stage of infection [14]. Exogenous contamination is more likely to occur if the hygiene conditions of the farm are poor, and in the presence of high environmental biocontamination, may be independent of the stage of infection of the animal [7,11].

Considering MAP’s ability to contaminate the food chain and the possible zoonotic implications, it is desirable to improve our knowledge concerning its survival in dairy products. To help bridge this gap, this study aims at evaluating the persistence of MAP in a specific type of raw goat milk cheese, known as “Robiola di Roccaverano”, along the manufacturing process using spiked milk. “Robiola di Roccaverano D.O.P.” is a soft cheese, made with raw goat milk or a mixture of goat and bovine milk, handcrafted in the hilly territory in the province of Asti, Piedmont, Italy.

## 2. Materials and Methods

### 2.1. Experimental Design

Different strains of MAP were used for the experimental inoculation of goat milk: ATCC 19698, which is a reference strain of bovine origin and a “field isolates cocktail”. Field isolates used for artificial contamination contained six different C-types, which were isolated from faeces or mesenteric lymph nodes of goats bred in seropositive farms located in Roccaverano. The ATCC strain was revitalized following the manufacturer’s instruction and then cultured on Herrold’s egg yolk medium that was supplemented with Mycobactin J (2 mg/L) (ID-Vet, Montpellier, France) (HEYM). The field isolates were cultured both on HEYM and on an automated culture system on liquid media (Versatrek^®^/ESP^®^) for the first isolation. Both field isolates and reference strains were subcultured in 7H9 Middlebrook broth (Becton Dickinson, Milan, Italy), supplemented with Mycobactin J (2 mg/L) and with OADC (Becton Dickinson).

From a starting solution of 10^9^ colony-forming units (CFU)/mL of MAP, two batches of raw goat milk (6 L each) were separately spiked with the ATCC strain (Batch A) and a mixture of field isolates (Batch B), obtaining a final concentration of 10^7^ CFU/mL. A total of six cheeses were produced, three from each curd, according to the manufacturer’s instructions. Furthermore, the milk used for this challenge test was previously tested by f57-qPCR [15], in order to verify the absence of natural MAP contamination.

### 2.2. Manufacturing Process

The cheese manufacturing process was reproduced in an experimental cheese factory according to the specific procedural guidelines named “Disciplinare di Produzione Robiola di Roccaverano” in order to obtain six cheese wheels (three for each batch), as described below.

Raw milk is heated to 20–22 °C and a natural lactic starter culture is added. After 4–8 h, when the pH is around 6.20–5.80, the rennet is added to induce natural coagulation and the acid curd is then delicately transferred into perforated moulds. It is left in the moulds for up to 48 h and turned regularly to speed up the extraction of the whey. The salting is dry on both sides of the product during the turning of the wheels. The fresh product is kept on steel grids in special rooms for at least three days at 15–20 °C, then it is placed in cold rooms at 4 °C. From the fourth day after the curd is placed in moulds, the robiola may be sold and it is considered refined from the tenth day. 

### 2.3. Sampling

Samples were taken in four stages: T0 (spiked milk), T1 (curd), T2 (cheese at 5 days), T3 (cheese at 10 days). Sampling was carried out twice, on both 5 mL of milk and 5 g of curd and cheese.

For the batch spiked with the ATCC strain, the microbiological parameters (lactic acid bacteria and mesophilic lactococci) and the chemical–physical parameters (pH and Aw) were also monitored at T0, T1, T2, and T3. 

### 2.4. Biomolecular Assays

Based on the data obtained by Hanifian [16], in which the quantification of MAP by cultural assay mirrored the quantification by f57-qPCR exactly, we preferred to use the latter for the monitoring of MAP load during cheese processing. Quantitative PCR was performed on samples taken in duplicate at T0, T1, T2 and T3 from both batches. DNA was extracted following a previously described protocol [15] and amplified with qPCR, targeting the F57 sequence according to Ricchi et al. [15,17]. All F57-qPCR reactions were performed on the CFX96 Touch Real-Time PCR Detection System (Biorad), in triplicates for each sample, in a volume of 20 μL of total master mix containing 300 nM of each primer, 6 nM of the probe and the positive internal control (TaqMan “Exogenous Internal Positive Control, Life Technologies) for the verification of the inhibition of the PCR reaction. For each run, at least one negative template control, one positive control, and one positive extraction control were included. The amplification involves 40 cycles composed of denaturation for 15 s at 95 °C followed by annealing and elongation for 60 s at 60 °C. Absolute MAP quantity was determined by calibration curves generated using plasmid standards, previously prepared at the Italian National Reference Centre for Paratuberculosis [18]. Each F57-qPCR was run with plasmid standard dilutions and the number of MAP was calculated using linear regression calculated by the cycles of quantification (Cq) obtained for each plasmid standard dilution.

### 2.5. Cultural, Physical and Chemical Assays

#### 2.5.1. MAP Cultural Assay

All samples were cultured on two selective media: Herrold’s egg yolk medium (HEYM) and Middlebrook 7H10 agar (7H10) (Becton Dickinson), and combined with PANTA (polymyxin, amphotericin B, nalidixic acid, trimethoprim, and azlocillin) (Becton Dickinson).

#### 2.5.2. LAB Cultural Assay

Lactic flora (lactobacilli) were counted in batch A samples according to the ISO 15214:1998.

#### 2.5.3. Mesophilic Lactococci Cultural Assay

Mesophilic lactococci were counted in batch A samples according to the ISO 15214:1998 with appropriate dilution on De Man Rogosa and Sharpe agar (MRS; Oxoid).

#### 2.5.4. Water Activity (Aw) and pH Assays

For batch A samples, the water activity (Aw) (ISO 21807:2004; ISO, 2004a) and the pH value (method MFHPB-03:2003) were determined by AcquaLab 4TE (Decagon Devices Inc., USA) and by pH meter micropH2001 (Crison, Barcelona, Spain), respectively.

### 2.6. Statistical Analysis

The results of qPCR were expressed as MAP cells per mL or g and were converted in log_10_. The non-parametric Friedman’s test for repeated measures analysis of variance (ANOVA) was performed to identify the significant effect of time on MAP survival, since data showed a non-normal distribution (Shapiro–Wilk test *p*-value > 0.05). Wilcoxon signed-rank tests was used as post hoc tests to identify the difference between time T0 and T3 time-points on MAP counts. In addition, the Dunnet’s test was used to compare each time point within the treatments with their respective T0. The Bonferroni correction was used to adjust for multiple hypothesis testing, such that *p*-values below 0.017 were considered statistically significant in the post hoc tests. All statistical analysis was performed using SAS version 4.

## 3. Results

All samples submitted to qPCR were quantifiable and the initial values (T0) were confirmed to be around 10^7^ MAP cells/mL for the ATCC strain, whereas the batch made with field isolates showed a lower value, consisting of around 10^6^ MAP cells/mL. Curd samples (T1) showed a load of around 10^6^ MAP cells/g, for both batches. In cheese sampled at 5 days of maturation (T2), we observed a decrease in the MAP load of about 2–3 logs compared to the curd. In fact, the loads resulted in 10^4^ MAP cells/g and 10^3^ MAP cells/g for the ATCC and for the field isolate batches, respectively. The qPCR performed on cheese samples at 10 days of maturation (T3) showed a constant decrease in the load, reaching concentrations of 10^2^ MAP cells/g and 10^1^ MAP cells/g for ATCC and the field isolates batches, respectively (Table 1). For each sample, MAP viability was confirmed by culture on HEYM and Middlebrook 7H10 agar. However, on the first medium it was not possible to perform a MAP count for both kind of batches (reference strain and field isolates) because of the presence of molds and lactic bacteria. Instead, on 7H10 agar, it was possible to count the MAP load on curd and cheese at 5 days for the ATCC strain and on milk and curd for the field strains. The results obtained were approximately one logarithm lower than the values reported with the qPCR (Table 1).

**Figure 1:**
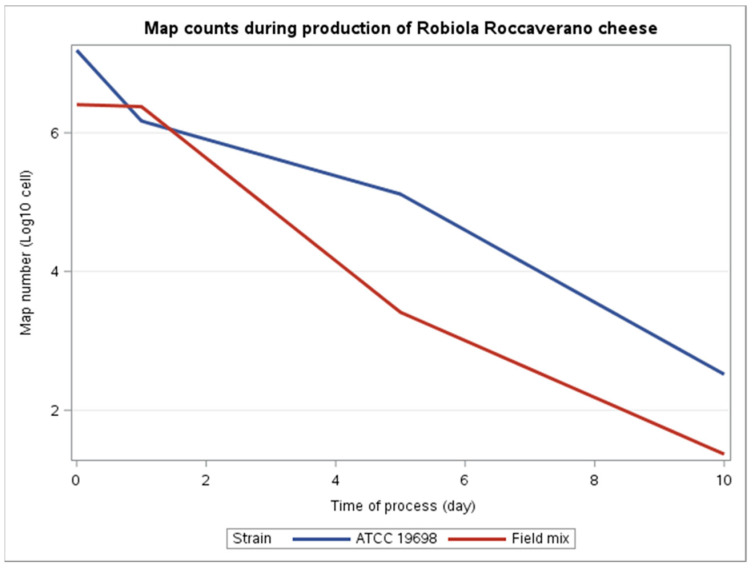
Variation of MAP count throughout the manufacturing and ripening of Robiola di Roccaverano cheese production as estimated by qPCR for ATCC 19698 strain and field strain mixture.

Despite the field isolates batch showing lower loads than ATCC, the difference between the two batches was not statistically significant (*p*-value = 0.6744). The reduction in MAP during the production process was statistically significant for both ATCC (median reduction of −4.67; *p*-value < 0.001) and the field isolates mix (median reduction of −5.04; *p*-value < 0.05) (Figure 1). 

The main changes in microbiological and physicochemical properties of cheese were recorded for the ATCC batch during the sampling times. Lactic acid bacteria (LAB) concentration was 4.89 CFU/mL in milk and raised during the cheesemaking process and the moulding phase until 9.23 CFU/g in the curd, although during the ripening phase there was a slight decrease in LAB concentration. The initial growth of LAB caused a rapid drop in the pH, starting from a pH of 6.21 in milk to a pH of 4.25 in the curd, whereas during the ripening phase, the pH value remained stable. Mesophilic lactococci concentration progressively increased during the cheesemaking process and during the ripening phase, whereas the activity water (aW) steadily decreased (Table 2).

**Figure 2:**
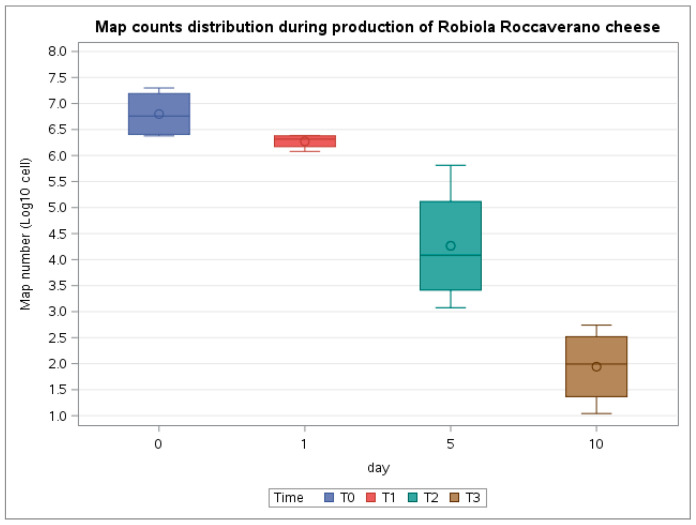
Boxplots displaying the MAP count distribution during the production of Robiola di Roccaverano cheese. The MAP count was performed by qPCR at four time points (T0–T3).

Figure 2 shows box plots with MAP concentration obtained by f57-qPCR at the four time points (T0–T3) for both batches. The mean difference between T0 and T1 was 0.53 (CI95% −0.75–1.80); however, the difference was not statistically significant. Meanwhile, the post-hoc analysis showed a reduction between T0 and T2 (mean reduction of 2.53; CI95% 1.26–3.81; *p*-value < 0.05) and between T0 and T3 (mean reduction of 4.86; CI95% 3.58–6.14; *p*-value < 0.05). A significant reduction was also found between T1 and T2 (mean reduction of 2.1; CI95% 0.73–3.29; *p*-value < 0.05) and between T2 and T3 (mean reduction of 2.32; CI95% 1.04–3.60; *p*-value < 0.05). 

Wilcoxon test showed a significant difference between MAP median value at T0 and MAP median value at T3 (*p*-value < 0.01).

## 4. Discussion

Biomolecular techniques are a valid alternative in MAP detection in milk and dairy product samples [19]. Quantitative PCR is more sensitive than bacterial culture, which tends to underestimate the real number of MAP cells present in the sample by approximately 1 or 2 log_10_ compared to qPCR [20]. This observation was also confirmed in our study. In fact, from the results obtained in the challenge test, in the few cases in which it was possible to obtain a bacterial count on the 7H10 medium, it was determined that the values found were approximately one logarithm lower than the values reported with qPCR. This appears due to both MAP tendency in forming cell aggregates, and to the death and/or possible dormancy of some MAP cells which remain detectable by qPCR but not by bacterial culture [21]. On the other hand, qPCR, counting the total number of cells present in the sample, regardless of their viability or not, tends to overestimate the actual mycobacteria concentration [15]. A similar situation was experienced by Galiero et al. [22], who detected MAP DNA in over half of goat and sheep cheeses by qPCR, whereas cultivation produced a positive result in only one case.

Overall, the experimental test carried out in our study showed a significant decrease in MAP DNA load from milk, curd and cheese for both reference and field isolates batches, with a similar behavior. The drop in MAP DNA can be explained by the decrease in pH values and by the proliferation of starter bacteria, which determine nutrient depletion and the production of antagonistic substances. This was the principal explanation provided in other studies about the reduction in different pathogens during the ripening period of cheeses [23,24,25]. Accordingly, the microbiological and physico-chemical parameters measured during the cheese manufacturing process for the ATCC batch showed a decrease in pH associated with an increase in lactic bacteria, particularly during the moulding phase. This mirrors what one study found on ultra-filtered white cheese, made with MAP spiked milk with an added starter culture. The addition of starter culture was suggested to be the main restrictive parameter affecting the survival of MAP, whereas the salt concentration did not accelerate MAP inactivation [16]. Notably, samples spiked with field isolates showed, on average, lower levels of MAP load than those spiked with the ATCC strain. Given the fact that both batches used the same starter culture and had the same productive process, the differences in the load between field isolates and the reference strain batches may be due to a difference in resistance to the manufacturing process. According to this, the laboratory-adapted strain ATCC 19698 has a higher thermal tolerance than that of the field isolates, as previously demonstrated [26]. 

In another study, the survival of MAP cells was monitored over a ripening period of 120 days in hard and semihard cheeses made with raw milk artificially contaminated with MAP [27]. In both cheeses, MAP load decreased steadily but slowly during cheese ripening. Similar results were obtained in another study aimed at investigating the survival of MAP during the manufacturing and ripening period of two Italian hard cheeses, Parmigiano Reggiano and Grana Padano, both made with raw bovine milk. In these products, the ripening phase largely deactivates MAP before the end of the minimum legal ripening period; the bacterium was undetectable with traditional cultural tests after two and three months of ripening for the cheeses obtained after spiking milk with field isolates and reference, respectively [28]. Therefore, the ripening period of cheese seems to be a determining factor in reducing the load of MAP; however, in our study, we cannot rely on this because “Robiola di Roccaverano” is a fresh cheese with a 10-day maturation period. A possible explanation for the reduction in the MAP load in a short period of time could be linked to the increased load of LAB populations, which in turn, after reaching a certain load, can start the production of bacteriocins, a wide class of molecules which showed variable killing activity against MAP [29]. One comprehensive study suggested that the in vitro killing activity of different bacteriocins against MAP varied according to their type [30]. In a more recent study, only nisin—a bacteriocin commonly proposed for the control of many foodborne pathogens—was evaluated, suggesting even a greater killing activity of nisin against MAP in respect to the former. The same study reported that this activity is linked to the ability of nisin to form pores in the cellular wall of MAP [31].

In addition to raw milk, pasteurized milk also presents a problem. Although the use of heat lowers the bacterial load, it does not guarantee that MAP has been completely eliminated [32,33]. The inefficiency of pasteurization on MAP inactivation is often correlated with a high starting charge in raw milk and is influenced by the tendency of mycobacterium to form “clumps” of bacterial cells (up to 10,000) so that the bacteria present in the central part would obviously be less exposed to thermal damage [34]. For these reasons, dairy products from pasteurized milk cheeses [35,36,37] may also pose a risk of infection by MAP due to the possible failure of this process to totally remove MAP cells [10,11]. 

In our study, MAP persistence in cheese during the ripening phase was probably related to the high initial contamination of the milk, estimated at 10^7^ CFU/mL, which is not realistic for natural milk contamination. In fact, to the best of our knowledge, even if no bibliographic data about the extent of MAP excretion into milk of goats are available, according to some studies on cattle, an average contamination of 100 CFU/mL [38] for clinical cows and around 2–8 CFU/50 mL for those in the asymptomatic phase [13] was reported. Assuming such a low milk excretion, even in goats, and the efficacy demonstrated in our study by the production process in reducing the initial bacterial load, it is possible to hypothesize a low risk of exposure by ingesting cheese produced from endogenously contaminated raw goat milk.

On the other hand, the faecal shedding of MAP by asymptomatically infected cattle or cows with apparent clinical disease has been determined to range from 10^2^ to 10^12^ CFU/g, leading to a consistent risk of contamination during milking for infected herds [7]. Therefore, it is not possible to exclude a consistent risk of exposure in cases of faecal contamination of goat milk given the high variability of MAP excretion levels with faeces in ruminants. For these reasons, the only way to reduce the risk of goat milk contaminated by MAP seems to be the adoption of control plans aimed at reducing the intra-herd prevalence of paratuberculosis, as recently suggested for the managing and breeding of other species [39].

## 5. Conclusions

In this study, we evaluated the persistence of MAP in a raw goat’s milk cheese through biomolecular assay. The reduction in the bacterial load suggests that the production process of “Robiola di Roccaverano” is able to inhibit bacterial survival and replication even if the milk used has not undergone any heat treatment and if the maturation period is considered short. 

At present, research on the persistence of MAP in goat milk cheese is scarce. Considering public concern about human exposure to MAP and the uncertainty of zoonotic potential, future studies should focus on the assessment of MAP contamination in milk and dairy products from small ruminants.

## Figures and Tables

**Table 1:** MAP load carried out in duplicate on 5 mL of milk and 5 g of curd and cheese by qPCR and by cultural assay on 7H10 agar during the cheese manufacturing process of Batch A and Batch B.

Time of Sampling	Product	PCR Assay(MAP Cells/mL for Milk or Map Cells/g for Curd and Cheese)		Cultural Assay(CFU/g for Curd and Cheese)	
		Batch A	Batch B	Batch A	Batch B
T0	Spiked Milk	1.99 × 10^7^ 1.21 × 10^7^	2.38 × 10^6^ 2.73 × 10^6^	n.d.^a^	1 × 10^5^ n.d. ^a^
T1	Curd	1.82 × 10^6^ 1.20 × 10^6^	2.34 × 10^6^ 2.43 × 10^6^	6 × 10^5^ 3 × 10^5^	6 × 10^5^ 1 × 10^5^
T2	Cheese (5 days)	2.62 × 10^4^ 6.50 × 10^5^	1.19 × 10^3^ 5.61 × 10^3^	5.5 × 10^4^ 3.5 × 10^4^	n.d.^a^
T3	Cheese (10 days)	1.97 × 10^2^ 5.5 × 10^2^	1.10 × 10^1^ 4.9 × 10^1^	n.d. ^a^	n.d. ^a^

^a^ n.d. not determined.

**Table 2:** Microbiological and physicochemical parameters during the cheese manufacturing process for Batch A.

Time of Sampling	Product (Batch A)	pH	aW	LAB ^b^	Mesophilic Lactococci ^b^
T0	Spiked Milk	6.21	n.d.^a^	4.89	3.2
T1	Curd	4.25	0.986	9.23	4.9
T2	Cheese (5 days)	4.25	0.983	7.78	5.2
T3	Cheese (10 days)	4.11	0.981	6.81	8.7

^a^ n.d. not determined; ^b^ Values are expressed in log_10_ CFU/mL or g.

## Data Availability

Not applicable.
